# Alzheimer’s Disease And Related Dementias Among Transfeminine Adults: A Cohort Study

**DOI:** 10.1093/geroni/igaf122.2397

**Published:** 2025-12-31

**Authors:** Ethan Cicero, Jace Flatt, Vin Tangpricha, Darios Getahun, Courtney McCracken, Michael Silverberg, Suma Vupputuri, Michael Goodman

**Affiliations:** Emory University, Atlanta, Georgia, United States; University of Nevada Las Vegas, Nevada, United States; Emory University, Atlanta, Georgia, United States; Kaiser Permanente Southern California, Pasadena, California, United States; Kaiser Permanente Georgia, Atlanta, Georgia, United States; Kaiser Permanente Northern California, Oakland, California, United States; Kaiser Permanente Mid-Atlantic States, Washington, District of Columbia, United States; Emory University, Atlanta, Georgia, United States

## Abstract

Understanding of Alzheimer’s disease and related dementias (ADRD) among transfeminine (TF) adults is limited. Kaiser electronic health records (01/2006–03/2023) were used to identify a cohort of 2,362 TF individuals aged 45+ enrolled in three integrated health systems. Each transgender individual was matched to 10 cisgender men (CM) and 10 cisgender women (CW) on birth year, race/ethnicity, study site, and enrollment at index date (first evidence of transgender status in TF cohort members). Enrollment time-adjusted odds ratios (aOR) and 95% confidence intervals (CI) were calculated to examine the odds of ADRD among TF adults compared to CM and CW referents. TF adults had a higher ADRD prevalence (1.8%) relative to CM (0.8%) and CW (0.9%). The aOR (95% CI) estimates of ADRD among TF were 1.61 (1.14–2.25) and 1.37 (0.98–1.91) relative to CM and CW, respectively. When restricting analyses to TF with evidence of gender-affirming hormone therapy (GAHT) and their referents, the associations were only slightly stronger than in the overall analysis with aOR (95% CI) estimates of 1.82 (1.20–2.75) and 1.29 (0.54–3.11) related to CM and CW, respectively. Results restricted to cohort members of color were similar, but imprecise due to small numbers of ADRD cases. TF adults have significantly higher odds of ADRD compared to CM and CW referents; this association does not appear to be attributable to GAHT, although hormone levels should be explored. Modifiable risk factors for ADRD should be examined to prevent or delay the onset of ADRD in transgender persons.

